# Supplementary material for: Antimicrobial Activities of Highly Bioavailable Organic Salts and Ionic Liquids from Fluoroquinolones
Source: Pharmaceutics. 2020 Jul 23;12(8):694. doi: 10.3390/pharmaceutics12080694 (PMC7464485; doi:10.3390/pharmaceutics12080694)
Supplement: Supplementary file 1 [file pharmaceutics-12-00694-s001.pdf]

pharmaceutics

MDPI

Supplementary Materials

Highly Bioavailable Organic Salts and Ionic Liquids from Fluoroquinolones Presenting Tailorable Antimicrobial Activities against Gram-Positive and Gram-Negative Bacteria

General remarks

Commercially available reagents were purchased from Aldrich, BDH-laboratory reagents, Alfa Aesar and Solchemar and were used as received. The solvents were purchased from Laborspirit and distilled before used. The basic anion-exchange resin Amberlite IRA-400-OH (ion-exchange capacity 1.4 eq.mL^{-1}) was purchased from Supelco. ^{1}H and ^{13}C NMR spectra of the final compounds were recorded on a Bruker AMX400 spectrometer in a 1:3 mixture of DMSO-d^6 and $CDCl_3$ (from Euriso-Top) at 298 K. Chemical shifts are reported dowfield in parts per million (ppm) from DMSO-d^6 reference. IR spectra were measured on a Perkin Elmer 683 from KBr matrixes of the compounds. Melting temperature (T_m) was determined by melting point apparatus (Stuart Scientific). DSC analysis was carried out using a TA Instruments Q-series TM Q2000 DSC with a refrigerated cooling system. The sample is continuously purged with 50 mL/min nitrogen flow. About 5 to 10 mg of each FQ-OSIL was crimped in an aluminum standard sample pan with pinhole lid. Elemental analysis were performed by Laboratório de Análises at REQUIMTE, Departamento de Química Faculdade de Ciências e Tecnologia (Monte de Caparica) using Thermo Finnigan-CE Elemental Analyser 1112 series.

Methods

All FQ-OSILs were prepared according to the following general procedure.

General procedure

The halide salt was dissolved in methanol and passed through an ion-exchange column Amberlite IRA-400-OH (5 eq., flux rate 0.133 mL/mL.min). The methanolic solution of the corresponding hydroxide salt was added dropwise to ciprofloxacin (1.2 eq) or norfloxacin (1.2 eq) completely dissolved in 2 M ammonia solution (50 mL/g). After stirring the mixture for 1 h, the solvent was evaporated in the rotavapor and the crude was recrystallized from a mixture of chloroform /methanol. Excess fluoroquinolone was filtered off, the solution was evaporated and the desired FQ-OSIL was dried in *vacuo* for 24h.

2-hydroxy-*N*,*N*,*N*-trimethylethanaminium 1-cyclopropyl-6-fluoro-4-oxo-7-(piperazin-1-yl)-1,4-dihydroquinoline-3-carboxylate [Ch][Cip]

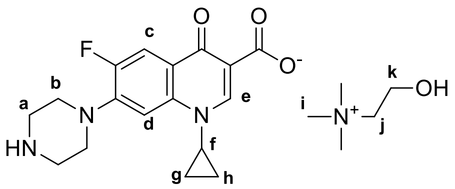

Using ciprofloxacin (360 mg, 1.086 mmol) and choline chloride (126 mg, 0.905 mmol), [Ch][Cip] was obtained in 96% yield as a pale yellow solid (357 mg). T_m = 111.2 ºC; ^{1}H NMR (400 MHz, DMSO-*d*^{6}/$CDCl_3$) δ 8.34 (s, 1H, H_e), 7.76 (d, J = 13.6 Hz, 1H, H_c), 7.29 (d, J = 7.1 Hz, 1H, H_d), 3.92 (br s, 2H, H_j), 3.54 (t, J = 4.7 Hz, 2H, H_k), 3.49 – 3.41 (m, 1H, H_f), 3.22 (s, 9H, H_i), 3.13 (t, J = 4.3 Hz, 4H, H_b), 2.93 (t, J = 4.3 Hz, 4H, H_a), 1.27 – 1.18, 1.06 – 0.97 (m, 2H each, H_g, H_h); ^{13}C NMR (101 MHz, DMSO-*d*^{6}/$CDCl_3$) δ 178.2, 167.7, 152.3 (d, J = 246.9 Hz), 145.0, 144.2 (d, J = 13.9 Hz), 138.2, 120.7, 111.3 (d, J = 21.8 Hz), 104.7, 67.2, 55.2, 53.2 (t, J = 3.7 Hz), 50.8, 45.3, 33.8, 7.5; IR (KBr): 3423, 1630, 1578, 1479, 1379, 1293, 1257, 942, 732 cm^{-1}; analysis calcd. for $C_{22}H_{31}FN_4O_4(H_2O)_{2.8}$: C, 54.49; H, 7.61; N, 11.55; found: C, 54.71; H, 7.92; N, 11.47.

2-hydroxy-*N*,*N*,*N*-trimethylethanaminium 1-ethyl-6-fluoro-4-oxo-7-(piperazin-1-yl)-1,4-dihydroquinoline-3-carboxylate, [Ch][Nor]

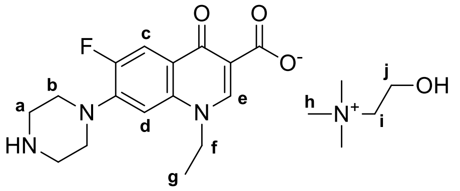

Using norfloxacin (600 mg, 1.885 mmol) and choline chloride (219 mg, 1.571 mmol), [Ch][Nor] was obtained in 93% yield as a pale yellow solid (617 mg). T_m = 94.5 ºC; T_g = 54.8 ºC; ^{1}H NMR (400 MHz, DMSO-*d*^{6}/$CDCl_3$) δ 8.40 (s, 1H, H_e), 7.81 (d, J = 13.7 Hz, 1H, H_c), 6.82 (d, J = 7.0 Hz, 1H, H_d), 4.24 (q, J = 7.0 Hz, 2H, H_f), 3.93 (br s, 2H, H_i), 3.53 (t, J = 4.7 Hz, 2H, H_j), 3.21 (s, 9H, H_h), 3.13 (t, J = 4.3 Hz, 4H, H_b), 2.92 (t, J = 4.3 Hz, 4H, H_a), 1.40 (t, J = 7.2 Hz, 3H, H_g); ^{13}C NMR (101 MHz, DMSO-*d*^{6}/$CDCl_3$) δ 174.4, 167.9, 152.2 (d, J = 247.0 Hz), 145.8, 144.5 (d, J = 12.7 Hz), 136.3, 121.5, 111.6 (d, J = 22.7 Hz), 103.9, 67.2, 55.3, 53.3 (t, J = 3.7 Hz), 50.8, 50.8, 47.9, 45.3, 14.0; IR (KBr): 3424, 1630, 1583, 1493, 1383, 1345, 1256, 1185, 1087, 929, 738 cm^{-1}; analysis calcd. for $C_{21}H_{31}FN_4O_4(H_2O)_{3.5}$: C, 51.95; H, 7.89; N, 11.54; found: C, 51.94; H, 7.84; N, 11.45.

1-ethyl-3-methyl-1*H*-imidazol-3-ium 1-cyclopropyl-6-fluoro-4-oxo-7-(piperazin-1-yl)-1,4-dihydroquinoline-3-carboxylate, [EMIM][Cip]

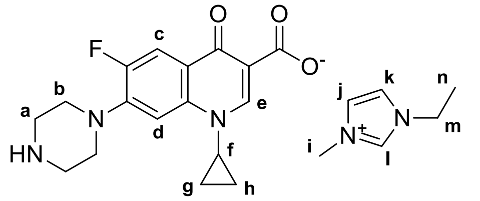

Using ciprofloxacin (436 mg, 1.316 mmol) and ethylmethylimidazolium bromide (210 mg, 1.097 mmol), [EMIM][Cip] was obtained in quantitative yield as a white solid (483 mg). T_m = 92.9 ºC; T_g = 36.7 ºC; T_g = 36.6 ºC; ^{1}H NMR (400 MHz, DMSO-*d*^{6}/$CDCl_3$) δ 8.46 (s, 1H, H_e), 7.80 (d, J = 13.4 Hz, 1H, H_c), 7.42 (br s, 1H, H_j), 7.37 (br s, 1H, H_k), 7.24 (d, J = 7.1 Hz, 1H, H_d), 4.33 (q, J = 7.3 Hz, 2H, H_m), 3.98 (s, 3H, H_i), 3.39 – 3.32 (m, 1H, H_f), 3.13 (t, J = 4.3 Hz, 4H, H_b), 2.96 (t, J = 4.3 Hz, 4H, H_a), 1.44 (t, J = 7.3 Hz, 3H, H_n), 1.23 – 1.16, 1.05 – 0.98 (m, 2H each, H_g, H_h) ppm; ^{13}C NMR (101 MHz, DMSO-*d*^{6}/$CDCl_3$) δ 174.5, 167.9, 152.2 (d, J = 245.6 Hz), 145.2, 144.1 (d, J = 16.0 Hz), 138.1, 123.0, 121.4, 121.1, 111.3 (d, J = 21.0 Hz), 104.6, 50.7, 50.3, 45.3, 44.0, 35.5, 15.1, 7.5 ppm; IR (KBr): 3424, 2852, 1629, 1577, 1491, 1381, 1294, 1257, 1171, 942, 733 cm^{-1}; anal. calcd. for $C_{23}H_{28}FN_5O_3(H_2O)_{6.0}$: C, 50.26; H, 7.34; N, 12.74; found: C, 50.32; H, 7.12; N, 12.51.

1-ethyl-3-methyl-1*H*-imidazol-3-ium 1-ethyl-6-fluoro-4-oxo-7-(piperazin-1-yl)-1,4-dihydroquinoline-3-carboxylate, [EMIM][Nor]

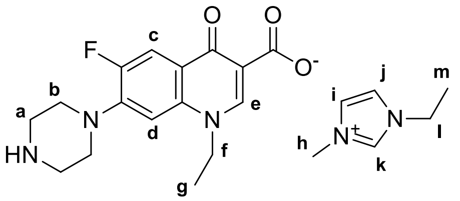

Using norfloxacin (600 mg, 1.885 mmol) and ethylmethylimidazolium bromide (300 mg, 1.571 mmol), [EMIM][Nor] was obtained in quantitative yield as a yellow solid (673 mg). T_f = 119.9 ºC; T_g = 64.6 ºC; ^{1}H NMR (400 MHz, DMSO-d^6/$CDCl_3$) δ 10.00 (br s, 1H, H_k), 8.60 (br s, 1H, H_e), 7.84 (d, J = 13.5 Hz, 1H, H_c), 7.60 (d, J = 1.6 Hz, 1H, H_i), 7.54 (d, J = 1.7 Hz, 1H, H_j), 6.87 (d, J = 7.0 Hz, 1H, H_d), 4.32 (q, J = 7.1 Hz, 2H, H_l), 4.27 (q, J = 7.3 Hz, 2H, H_f), 3.92 (s, 3H, H_h), 3.16 (t, J = 3.8 Hz, 4H, H_b), 2.97 (t, J = 3.8 Hz, 4H, H_a), 1.44 (t, J = 7.3 Hz, 3H, H_g), 1.43 (t, J = 7.1 Hz, 3H, H_m); ^{13}C NMR (101 MHz, DMSO-d^6/$CDCl_3$) δ 174.4, 167.7, 152.1 (d, J = 247.3 Hz), 146.0, 144.4 (d, J = 12.7 Hz), 136.2, 123.0, 121.7, 121.3, 111.6 (d, J = 22.3 Hz), 103.9, 50.8, 48.6, 47.8, 45.3, 44.0, 35.5, 15.2, 14.0 ppm; IR (KBr): 3423, 2989, 2852, 1628, 1581, 1492, 1389, 1255, 1170, 931, 824, 738 cm^{-1}; analysis calcd. for $C_{22}H_{28}FN_5O_3(H_2O)_{6.4}$: C, 48.50; H, 7.55; N, 12.86; found: C, 48.46; H, 7.24; N, 12.60.

1-(2-hydroxyethyl)-3-methyl-1*H*-imidazol-3-ium 1-cyclopropyl-6-fluoro-4-oxo-7-(piperazin-1-yl)-1,4-dihydroquinoline-3-carboxylate, [C2OHMIM][Cip]

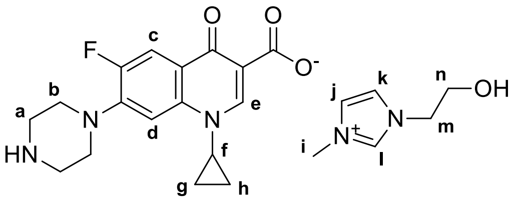

Using ciprofloxacin (546 mg, 1.646 mmol) and hydroxyethylmethylimidazolium chloride (223 mg, 1.372 mmol), [C2OHMIM][Cip] was obtained in quantitative yield as a white solid (627 mg). T_m = 111.6 ºC; T_g = 49.5 ºC; ^{1}H NMR (400 MHz, DMSO-d^6/$CDCl_3$) δ 10.34 (br s, 1H, H_l), 8.42 (br s, 1H, H_e), 7.82 (d, J = 13.5 Hz, 1H, H_c), 7.46 (d, J = 1.7 Hz, 1H, H_j), 7.32 (d, J = 1.7 Hz, 1H, H_k), 7.24 (d, J = 7.2 Hz, 1H, H_d), 4.40 (t, J = 4.9 Hz, 2H, H_m), 3.94 (s, 3H, H_i), 3.82 (t, J = 4.9 Hz, 2H, H_n), 3.37 (quint, J = 3.4 Hz, 1H, H_f), 3.14 (t, J = 4.4 Hz, 4H, H_b), 2.97 (t, J = 4.4 Hz, 4H, H_a), 1.25 – 1.17, 1.06 – 0.97 (m, 2H each, H_g, H_h); ^{13}C NMR (101 MHz, DMSO-d^6/$CDCl_3$) δ 174.2, 168.0, 152.2 (d, J = 244.3 Hz), 145.0, 144.0 (d, J = 18.8 Hz), 138.1, 122.7, 122.4, 121.2, 111.3 (d, J = 22.5 Hz), 104.7, 59.3, 51.3, 50.8, 45.3, 35.4, 7.46; IR (KBr): 3423, 2964, 2896, 2851, 1628, 1578, 1480, 1381, 1294, 1256, 1170, 941, 825, 733 cm^{-1}; analysis calcd. for $C_{23}H_{28}FN_5O_4(H_2O)_{5.0}$: C, 50.45; H, 6.99; N, 12.79; found: C, 50.55; H, 6.94; N, 12.68.

1-(2-hydroxyethyl)-3-methyl-1*H*-imidazol-3-ium 1-ethyl-6-fluoro-4-oxo-7-(piperazin-1-yl)-1,4-dihydroquinoline-3-carboxylate, [C2OHMIM][Nor]

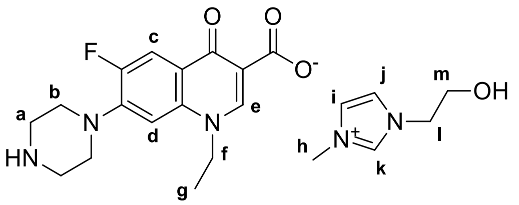

Using norfloxacin (546 mg, 1.714 mmol) and hydroxyethylmethylimidazolium chloride (232 mg, 1.428 mmol), [C2OHMIM][Cip] was obtained in quantitative yield as a yellow paste (634 mg). T_g = 41.1 ºC; ^{1}H NMR (400 MHz, DMSO-d^6/$CDCl_3$) δ 10.00 (br s, 1H, H_k), 8.44 (br s, 1H, H_e), 7.81 (d, J = 13.5 Hz, 1H, H_c), 7.67 (br s, 1H, H_i), 7.56 (br s, 1H, H_j), 6.86 (d, J = 6.9 Hz, 1H, H_d), 4.36 (t, J = 4.9 Hz, 2H, H_l), 4.27 (q, J = 7.1 Hz, 2H, H_f), 3.92 (s, 3H, H_h), 3.76 (t, J = 4.9 Hz, 2H, H_m), 3.13 (t, J = 4.0 Hz, 4H, H_b), 2.92 (t, J = 4.0 Hz, 4H, H_a), 1.40 (t, J = 7.1 Hz, 3H, H_g) ppm; ^{13}C NMR (101 MHz, DMSO-d^6/$CDCl_3$) δ 174.5, 168.3, 152.2 (d, J = 247.4 Hz), 145.9, 144.5 (d, J = 10.6 Hz), 136.2, 122.4, 122.3, 121.8, 111.7 (d, J =

22.5 Hz), 103.8, 78.3, 59.4, 51.4, 50.8, 47.9, 45.3, 35.5, 14.0 ppm; IR (KBr) 3417, 2990, 2852, 1627, 1581, 1492, 1389, 1341, 1255, 1169, 1066, 931, 825, 737 cm^{-1}; analysis calcd. for $C_{22}H_{28}FN_5O_4(H_2O)_{4.6}$): C, 50.01; H, 7.10; N, 13.25; found C, 50.10; H, 7.14; N, 12.99.

1-(2-hydroxyethyl)-2,3-dimethyl-1*H*-imidazol-3-ium 1-cyclopropyl-6-fluoro-4-oxo-7-(piperazin-1-yl)-1,4-dihydroquinoline-3-carboxylate, [C_2OHDMIM][Cip]

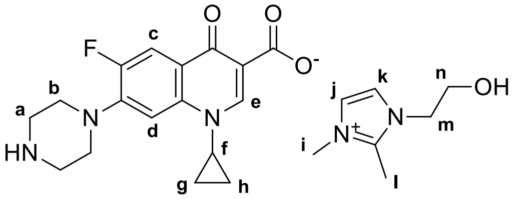

Using ciprofloxacin (500 mg, 1.509 mmol) and 1-hydroxyethyl-2,3-dimethylimidazolium chloride (222 mg, 1.257 mmol), [C_2OHDMIM][Cip] was obtained in quantitative yield as a white solid (718 mg). T_m = 192.1 ºC; ^{1}H NMR (400 MHz, DMSO-*d*^{6}/$CDCl_3$) δ 8.36 (br s, 1H, H_e), 7.77 (d, J = 13.9 Hz, 1H, H_c), 7.60 (d, J = 1.7 Hz, 1H, H_j), 7.43 (d, J = 1.7 Hz, 1H, H_k), 7.26 (d, J = 6.9 Hz, 1H, H_d), 4.27 (t, J = 4.5 Hz, 2H, H_m), 3.77 (t, J = 4.5 Hz, 2H, H_n), 3.76 (s, 3H, H_i), 3.58–3.49 (m, 1H, H_f), 3.14 (t, J = 4.2 Hz, 4H, H_b), 2.94 (t, J = 4.2 Hz, 4H, H_a), 2.64 (s, 3H, H_l), 1.28 – 1.15, 1.07 – 0.96 (m, 2H each, H_g, H_h) ppm; ^{13}C NMR (101 MHz, DMSO-*d*^{6}/$CDCl_3$) δ 174.2, 168.1, 152.3 (d, J = 249.9 Hz), 144.9, 144.2, 144.0 (d, J = 12.0 Hz), 138.1, 121.8, 121.5, 121.3, 111.5 (d, J = 22.7 Hz), 104.5, 59.6, 50.7, 45.3, 34.6, 9.5, 7.5; IR (KBr): 3424, 2897, 2853, 1631, 1581, 1482, 1383, 1296, 1258, 1182, 943, 827, 734 cm^{-1}; analysis calcd. for $C_{24}H_{30}FN_5O_4(H_2O)_{4.5}$: C, 52.16; H, 7.11; N, 12.67; found: C, 52.13; H, 7.14; N, 12.74.

1-(2-hydroxyethyl)-2,3-dimethyl-1*H*-imidazol-3-ium 1-ethyl-6-fluoro-4-oxo-7-(piperazin-1-yl)-1,4-dihydroquinoline-3-carboxylate, [C_2OHDMIM][Nor]

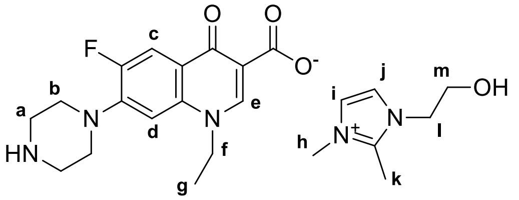

Using norfloxacin (474 mg, 1.508 mmol) and 1-hydroxyethyl-2,3-dimethylimidazolium chloride (222 mg, 1.257 mmol), [C_2OHDMIM][Nor] was obtained in quantitative yield as a white solid (576 mg). T_f = 121.8 ºC, T_g = 65.1 ºC; ^{1}H NMR (400 MHz, DMSO-*d*^{6}/$CDCl_3$) δ 8.36 (br s, 1H, H_e), 7.88 – 7.80 (m, 1H, H_c), 7.59 (d, J = 1.9 Hz, 1H, H_i), 7.41 (d, J = 1.9 Hz, 1H, H_j), 6.77 (d, J = 7.0 Hz, 1H, H_d), 4.27 (t, J = 4.9 Hz, 2H, H_l), 4.19 (q, J = 6.9 Hz, 2H, H_f), 3.78 (t, J = 4.9 Hz, 2H, H_m), 3.75 (s, 3H, H_h), 3.12 (t, J = 4.2 Hz, 4H, H_b), 2.94 (t, J = 4.2 Hz, 4H, H_a), 2.66 – 2.60 (m, 3H, H_k), 1.40 (t, J = 6.9 Hz, 3H, H_g); ^{13}C NMR (101 MHz, DMSO-*d*^{6}/$CDCl_3$) δ 175.3, 167.0, 152.6 (d, J = 249.3 Hz), 146.6, 145.2 (d, J = 16.0 Hz), 144.3, 136.6, 121.9, 121.4, 120.5, 111.5 (d, J = 22.9 Hz), 104.1, 59.6, 50.6, 50.5, 48.4, 45.2, 34.6, 14.0; IR (KBr): 3418, 2989, 2854, 1629, 1583, 1493, 1389, 1254, 1184, 931, 825 cm^{-1}; analysis calcd. for $C_{23}H_{30}FN_5O_4(H_2O)_{5.9}$: C, 48.82; H, 7.45; N, 12.38; found: C, 48.75; H, 7.19; N, 12.18.

1-(2-methoxyethyl)-3-methyl-1*H*-imidazol-3-ium 1-cyclopropyl-6-fluoro-4-oxo-7-(piperazin-1-yl)-1,4-dihydroquinoline-3-carboxylate, [C_3OMIM][Cip]

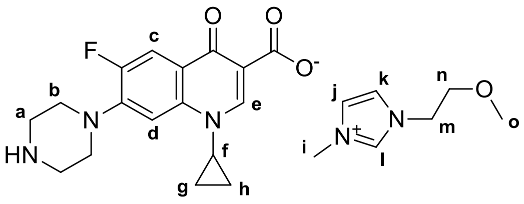

Using ciprofloxacin (500 mg, 1.509 mmol) and 1-(2-methoxyethyl)-3-methylimidazolium chloride (222 mg, 1.257 mmol), [C_3OMIM][Cip] was obtained in quantitative yield as a white solid (719 mg). T_m = 112.4 °C; T_g = 46.4 °C; ^{1}H NMR (400 MHz, DMSO-*d*^{6}/$CDCl_3$) δ 10.45 (br s, 1H, Hl), 8.44 (br s, 1H, He), 7.87–7.75 (m, 1H, Hc), 7.48 (d, J = 1.6 Hz, 1H, Hj), 7.45 (d, J = 1.6 Hz, 1H, Hk), 7.26 (d, J

= 6.6 Hz, 1H, Hd), 4.49 (t, J = 4.7 Hz, 2H, Hm), 3.97 (s, 3H, Hi), 3.66 (t, J = 4.7 Hz, 2H, Hn), 3.40 (quint, J = 3.8 Hz, 1H, Hf), 3.24 (s, 1H, Ho), 3.13 (t, J = 4.5 Hz, 4H, H_b), 2.95 (t, J = 4.5 Hz, 4H, H_a), 1.25–1.17, 1.06–0.98 (m, 2H each, H_g, H_h) ppm; ^{13}C NMR (101 MHz, DMSO-d^6/$CDCl_3$) δ 174.6, 167.7, 152.3 (d, J = 249.1 Hz), 145.4, 144.2 (d, J = 11.3 Hz), 138.2, 122.7, 122.3, 111.3 (d, J = 23.1 Hz), 104.6, 69.8, 58.1, 50.7, 48.6, 45.3, 35.5, 7.5 ppm; IR (KBr): 3423, 2958, 2900, 2847, 1630, 1577, 1479, 1379, 1293, 1257, 1174, 941, 825, 733 cm^{-1}; analysis calcd. for $C_{24}H_{30}FN_5O_4(H_2O)_{4.5}$: C, 52.16; H, 7.11; N, 12.67; found: C, 52.24; H, 7.04; N, 12.66.

1-(2-methoxyethyl)-3-methyl-1*H*-imidazol-3-ium 1-ethyl-6-fluoro-4-oxo-7-(piperazin-1-yl)-1,4-dihydroquinoline-3-carboxylate, [C₃OMIM][Nor]

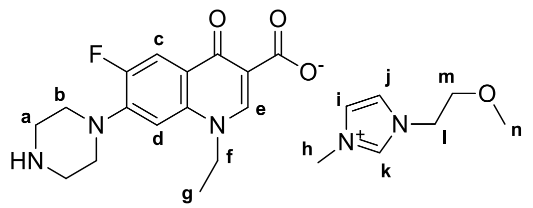

Using norfloxacin (600 mg, 1.885 mmol) and 1-(2-methoxyethyl)-3-methylimidazolium chloride (277 mg, 1.571 mmol), [C_3OMIM][Nor] was obtained in quantitative yield a white paste (721 mg). T_g = 44.1 °C; ^{1}H NMR (400 MHz, DMSO-d^6/$CDCl_3$) δ 9.98 (br s, 1H, H_k), 8.58 (br s, 1H, H_e), 7.83 (d, J = 13.5 Hz, 1H, H_c), 7.57 (d, J = 1.7 Hz, 1H, H_i), 7.54 (d, J = 1.7 Hz, 1H, H_j), 6.86 (d, J = 6.5 Hz, 1H, H_d), 4.44 (t, J = 4.4 Hz, 2H, H_l), 4.31 (q, J = 6.8 Hz, 2H, H_f), 3.94 (s, 3H, H_h), 3.66 (t, J = 4.4 Hz, 2H, H_m), 3.25 (s, 3H, H_n), 3.16 (t, J = 4.4 Hz, 4H, H_b), 2.93 (t, J = 4.4 Hz, 4H, H_a), 1.43 (t, J = 6.8 Hz, 3H, H_g) ppm; ^{13}C NMR (101 MHz, DMSO-d^6/$CDCl_3$) δ 174.5, 168.0, 152.1 (d, J = 247.6 Hz), 145.8, 144.4 (d, J = 10.7 Hz), 144.3, 138.9, 136.1, 122.5, 122.3, 121.9, 111.6 (d, J = 22.4 Hz), 103.6, 70.0, 58.1, 50.8, 50.7, 48.6, 45.3, 35.6, 13.9 ppm; IR (KBr) 3423, 2993, 2946, 2905, 2842, 1629, 1579, 1492, 1381, 1256, 1172, 930, 829, 738 cm^{-1} ; analysis calcd. for $C_{23}H_{30}FN_5O_4(H_2O)_{7.7}$: C, 46.18; H, 7.65; N, 11.71; found: C, 46.16; H, 7.20; N, 11.54.

Spectra

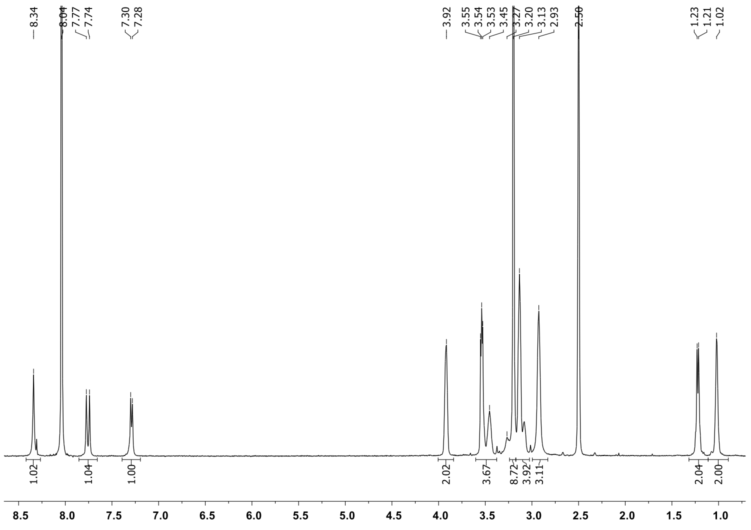

Figure S1. H NMR spectrum of [Ch][Cip] in DMSO-d^6/$CDCl_3$.

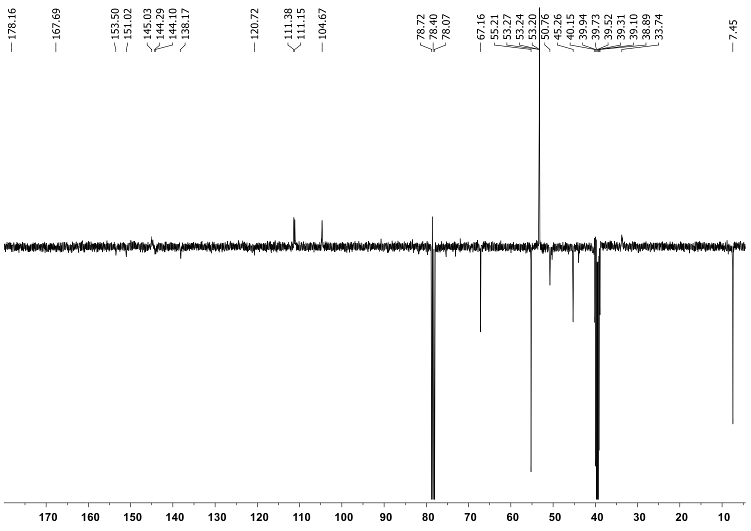

Figure S1 ^{13}C NMR (APT) spectrum of [Ch][Cip] in DMSO-d^6/$CDCl_3$.

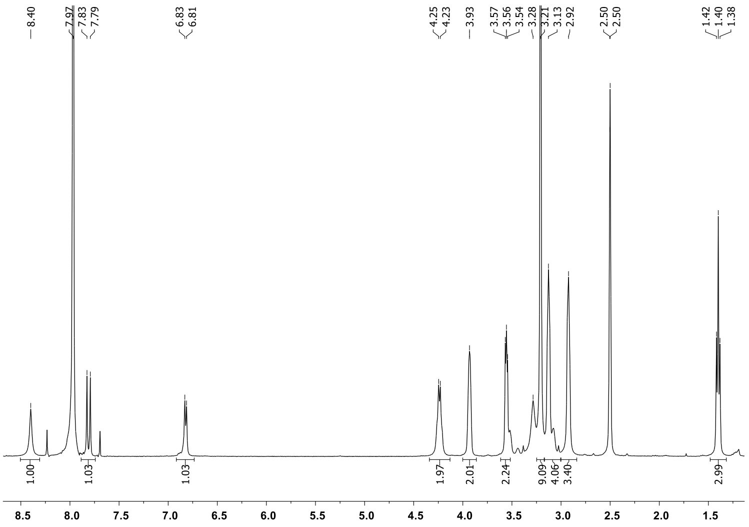

Figure S2 ^{1}H NMR spectrum of [Ch][Nor] in DMSO-*d*^{6}/$CDCl_3$.

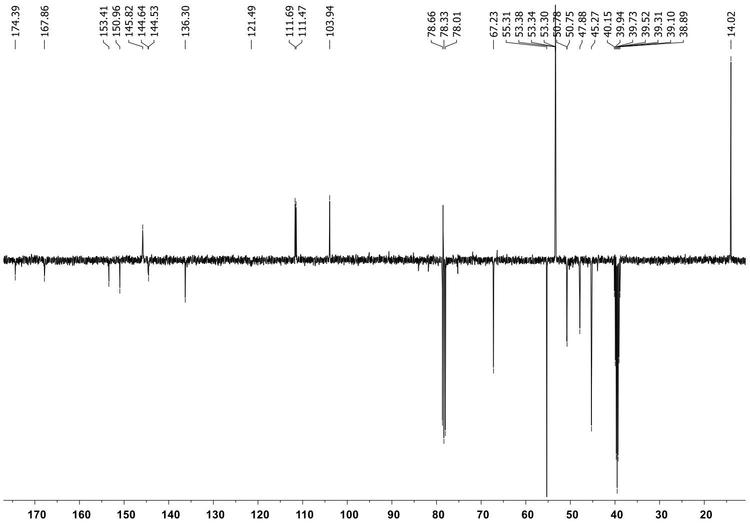

Figure S3 ^{13}C NMR spectrum of [Ch][Nor] in DMSO-*d*^{6}/$CDCl_3$.

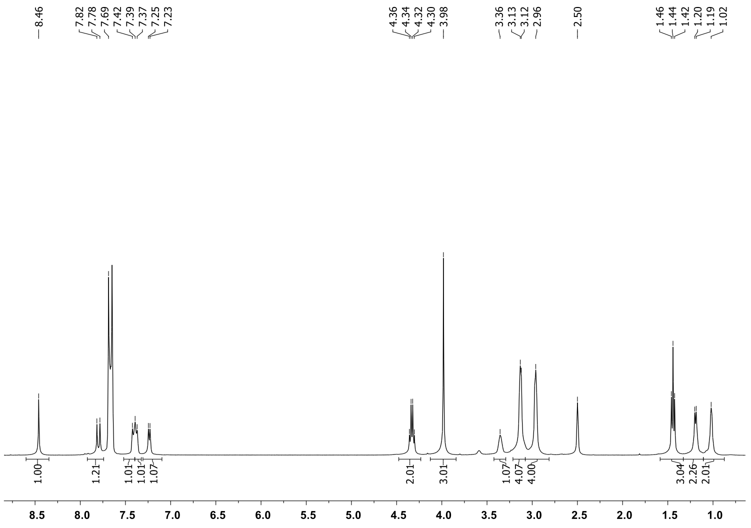

Figure S4 ^{1}H NMR spectrum of [EMIM][Cip] in DMSO-d^{6}/$CDCl_3$.

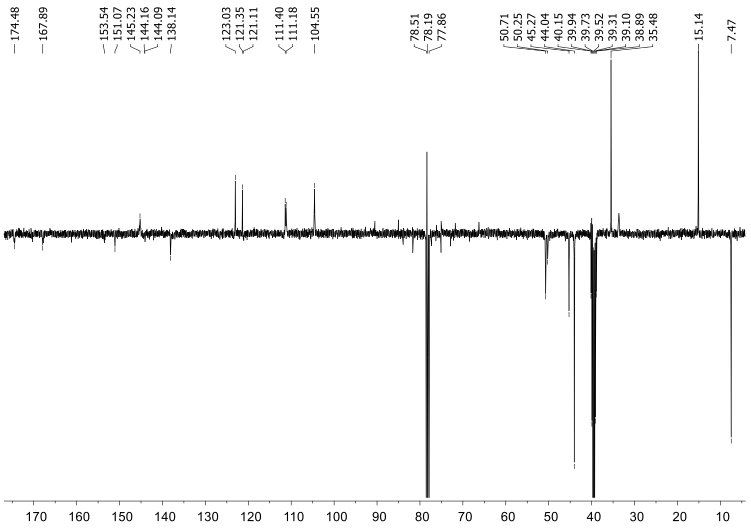

Figure S6 ^{13}C NMR spectrum of [EMIM][Cip] in DMSO-d^{6}/$CDCl_3$.

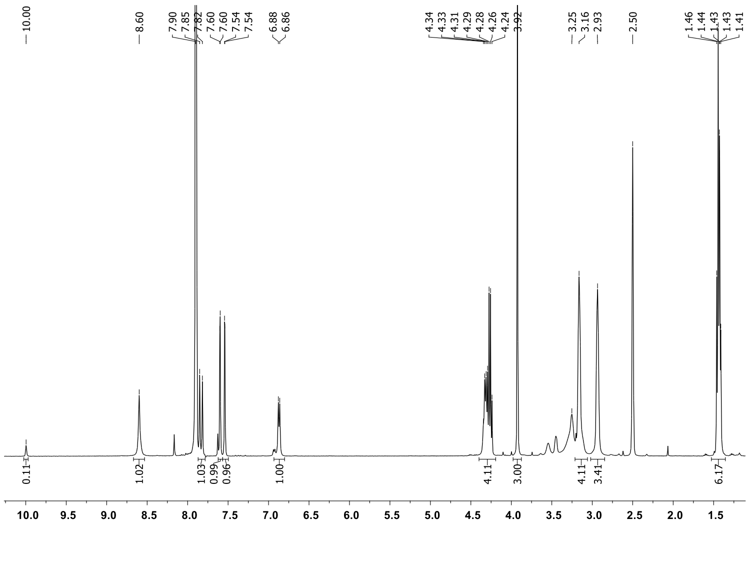

Figure S7 ^{1}H NMR spectrum of [EMIM][Nor] in DMSO-*d*6/CDCl3.

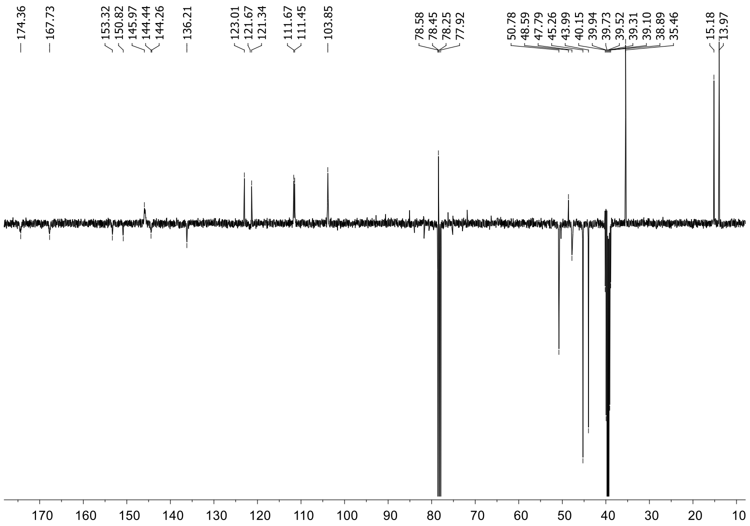

Figure S8 ^{13}C NMR spectrum of [EMIM][Nor] in DMSO-*d*6/CDCl3.

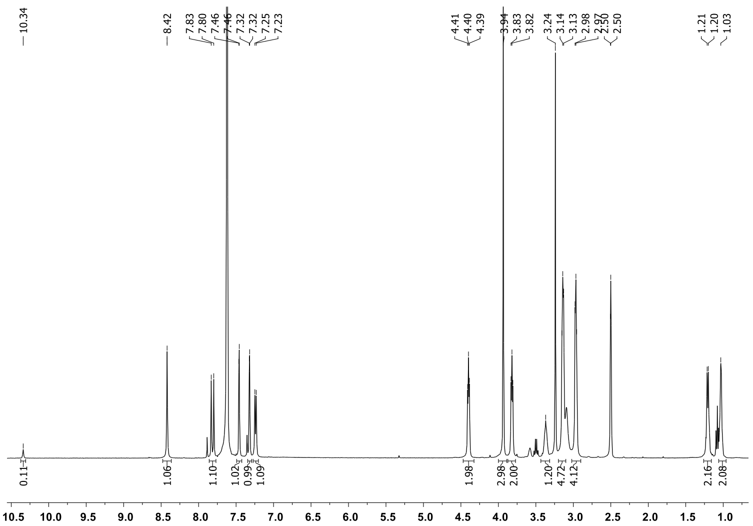

Figure S9 ^{1}H NMR spectrum of [C_2OHMIM][Cip] in DMSO-*d*6/CDCl3.

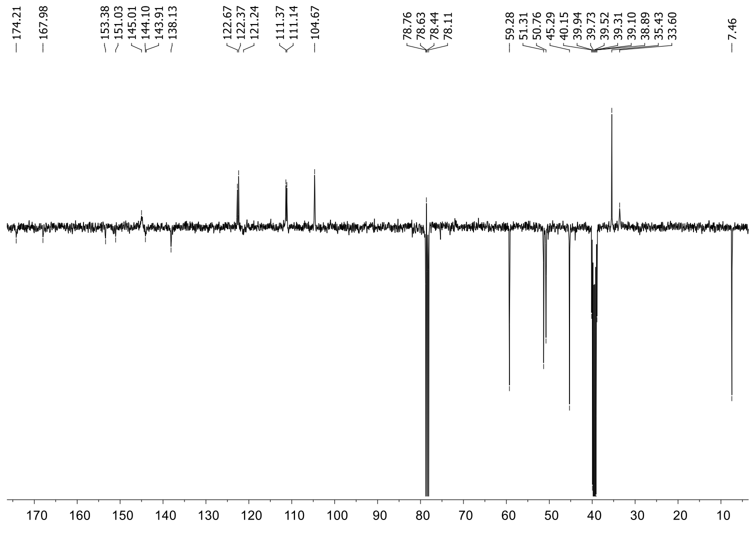

Figure S10 ^{13}C NMR spectrum of [C$_2$OHMIM][Cip] in DMSO-*d*^{6}/CDCl$_3$.

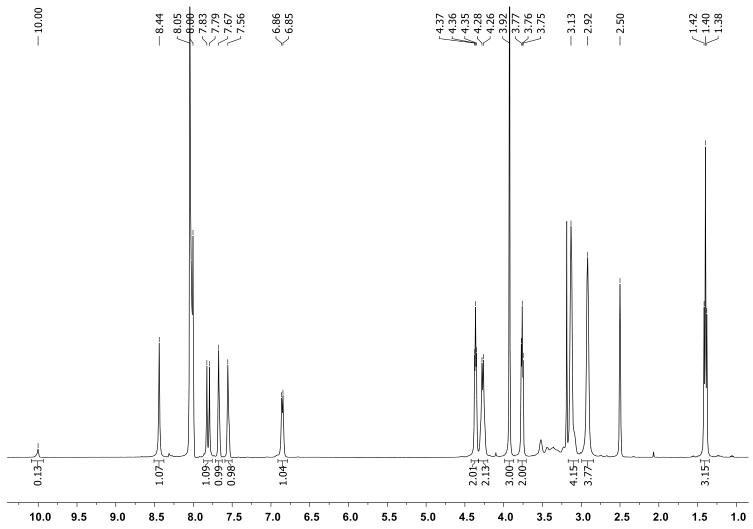

Figure S11 ^{1}H NMR spectrum of [C$_2$OHMIM][Nor] in DMSO-*d*^{6}/CDCl$_3$.

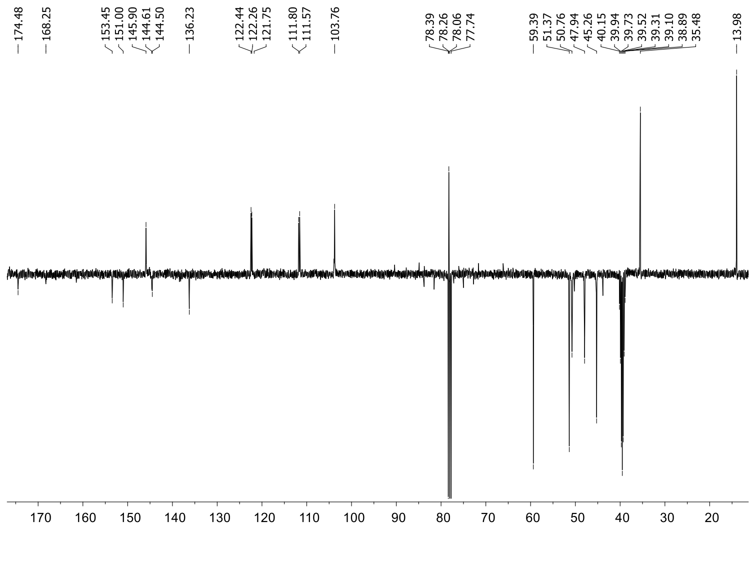

Figure S12 ^{13}C NMR spectrum of [C2OHMIM][Nor] in DMSO-d^6/CDCl$_3$.

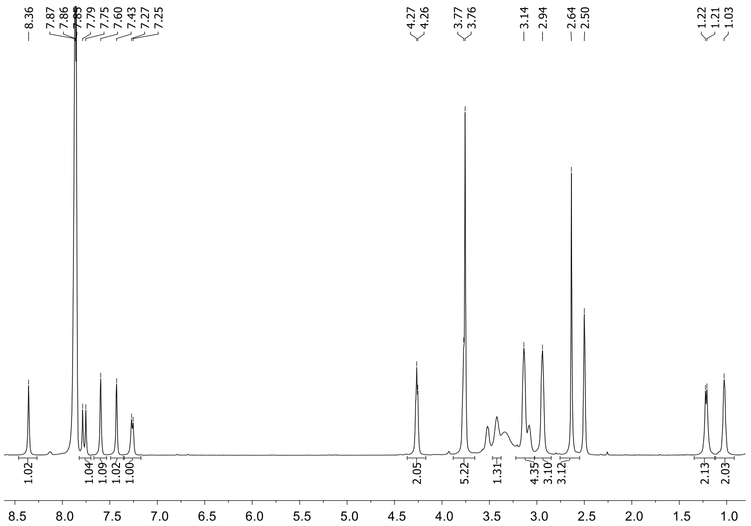

Figure S13 ^{1}H NMR spectrum of [C2OHDMIM][Cip] in DMSO-d^6/CDCl$_3$.

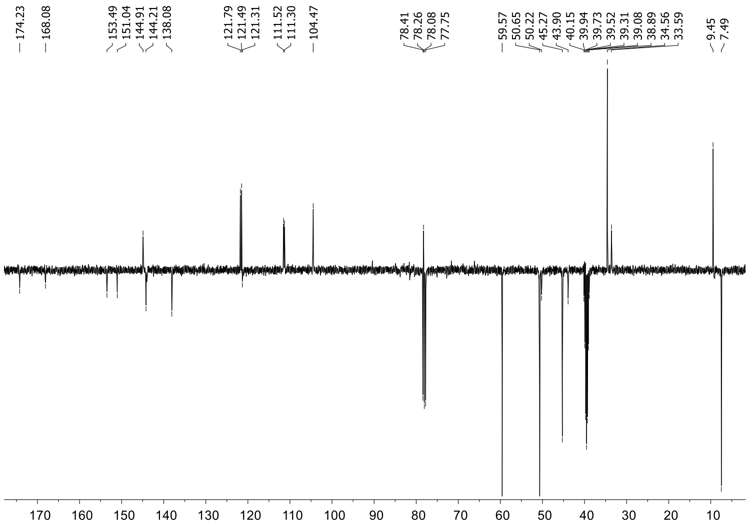

Figure S14 ^{13}C NMR spectrum of [C2OHDMIM][Cip] in DMSO-d^6/CDCl$_3$.

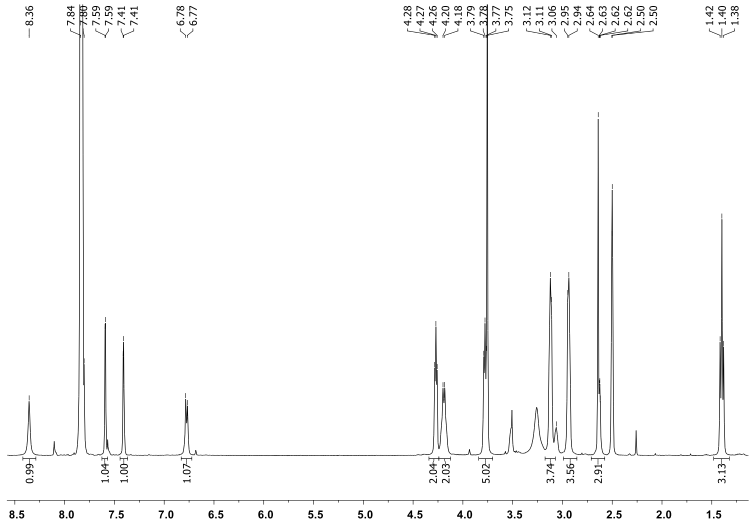

Figure S15 ^{1}H NMR spectrum of [C2OHDMIM][Nor] in DMSO-*d*6/CDCl3.

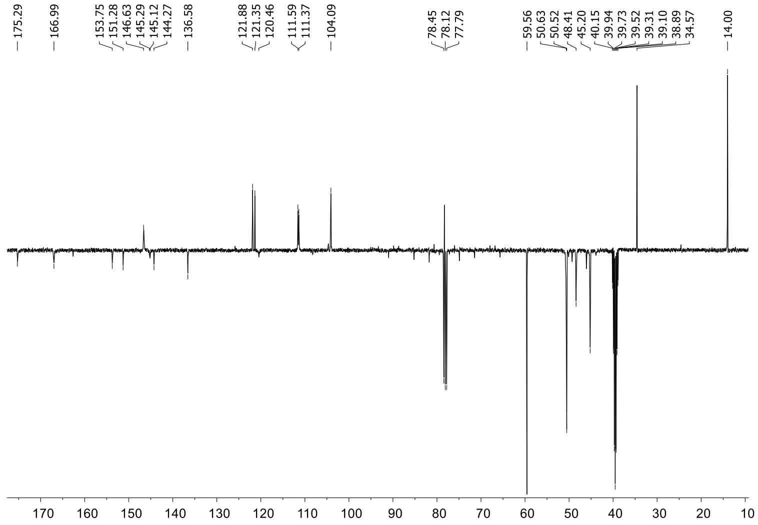

Figure S16 ^{13}C NMR APT spectrum of [C2OHDMIM][Nor] in DMSO-*d*6/CDCl3.

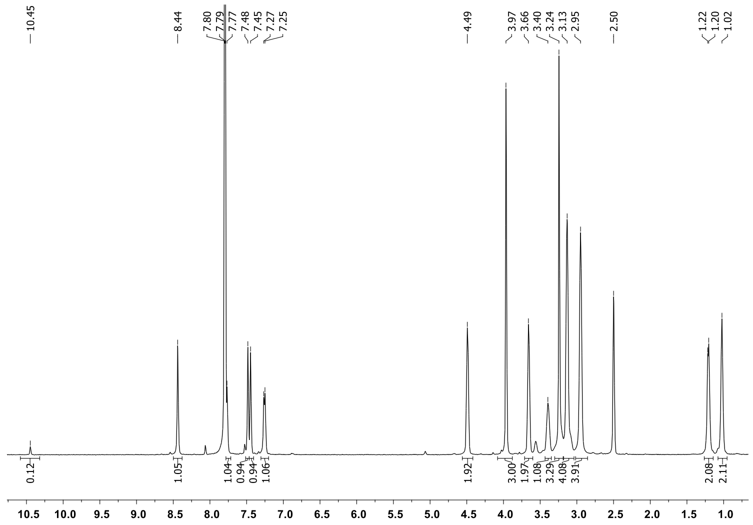

Figure S17 ^{1}H NMR spectrum of [C$_3$OMIM][Cip] in DMSO-*d*^{6}/CDCl$_3$.

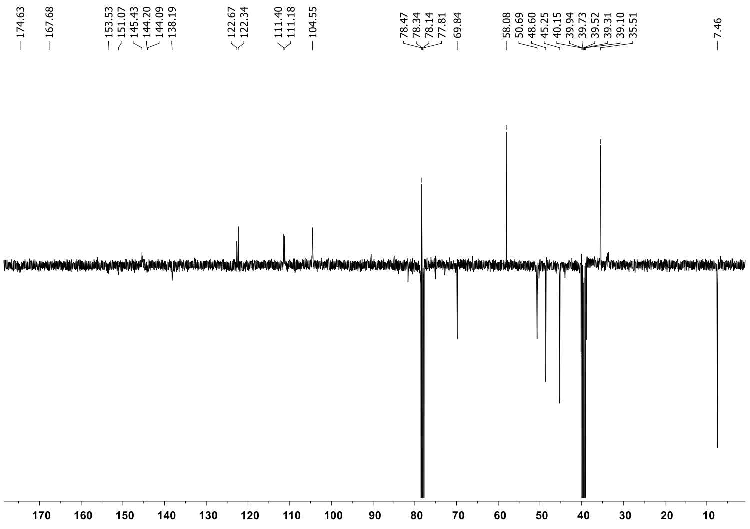

Figure S18 ^{13}C NMR spectrum of [C$_3$OMIM][Cip] in DMSO-*d*^{6}/CDCl$_3$.

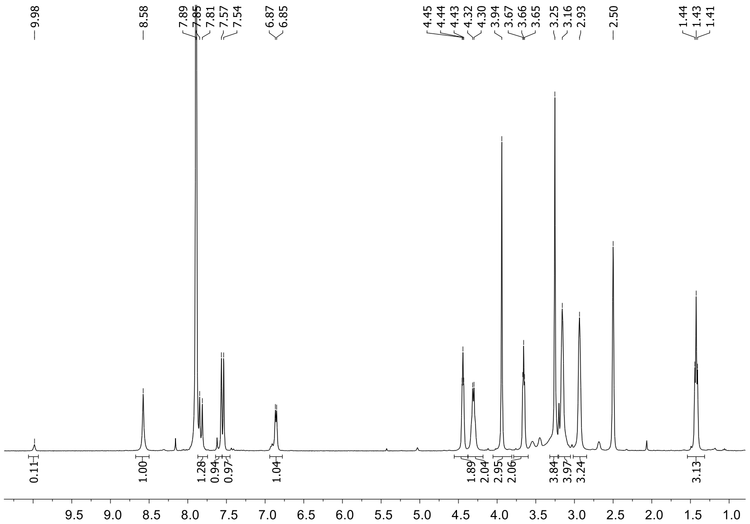

Figure S19 ^{1}H NMR spectrum of [C$_3$OMIM][Nor] in DMSO-*d*^{6}/CDCl$_3$.

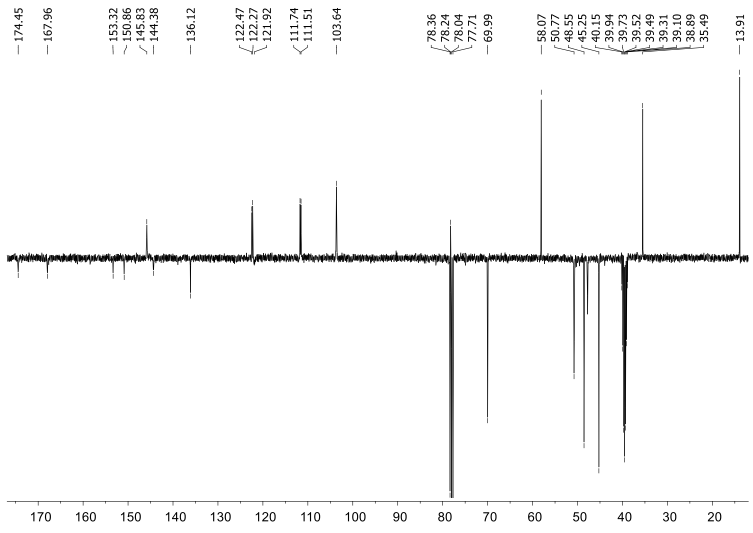

Figure S20 ^{13}C NMR APT spectrum of [C_3OMIM][Nor] in DMSO-d^6/$CDCl_3$.

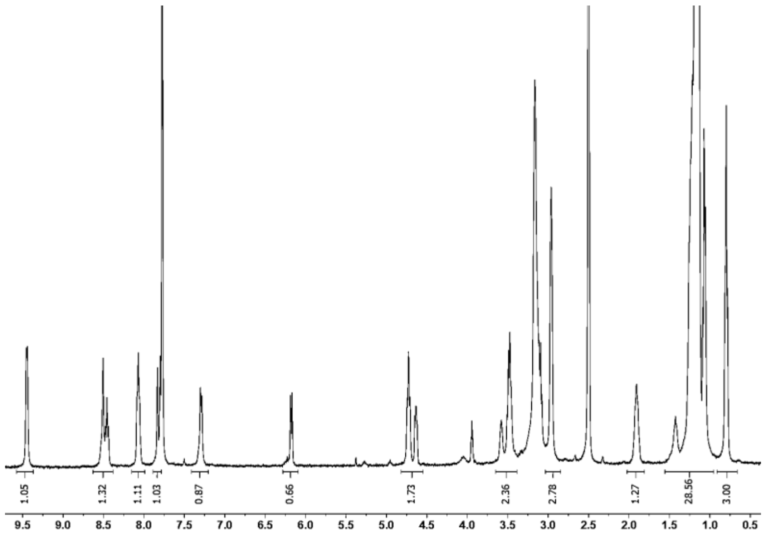

Figure S21 ^{1}H NMR spectrum of [C_{16}Py][Cip] in DMSO-d^6/$CDCl_3$.

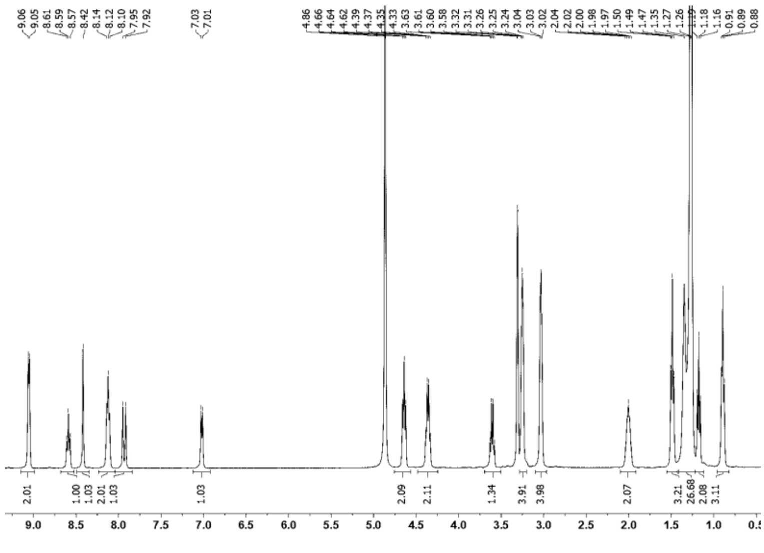

Figure S22 ^{1}H NMR spectrum of [C_{16}Py][Nor] in DMSO-d^6/$CDCl_3$.

FTIR spectra

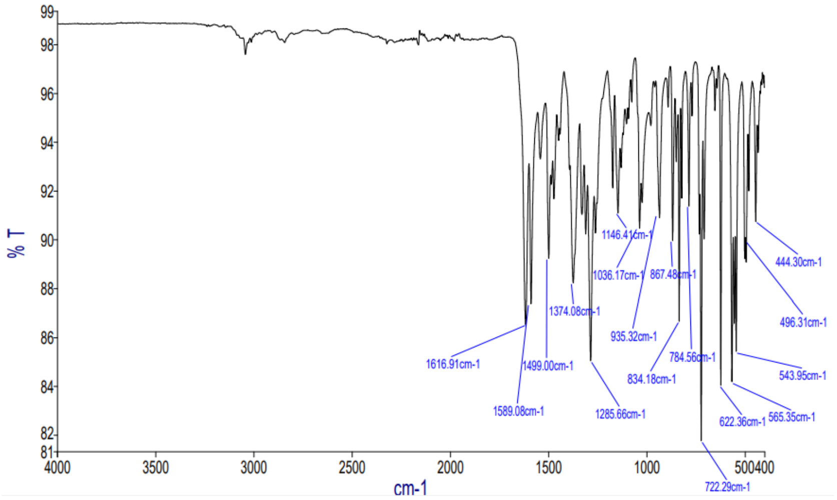

Figure S23 FTIR spectrum of ciprofloxacin in ATR mode.

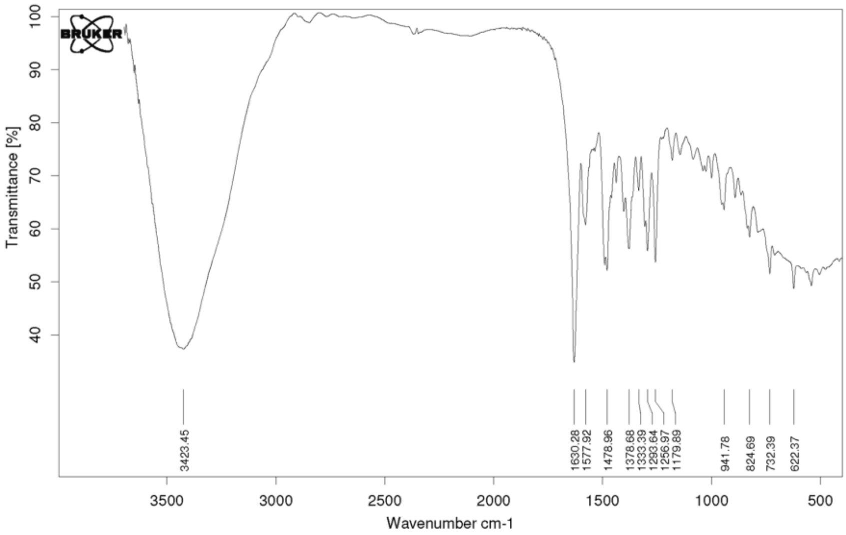

Figure S24 FTIR spectrum of [Ch][Cip] in KBr matrix.

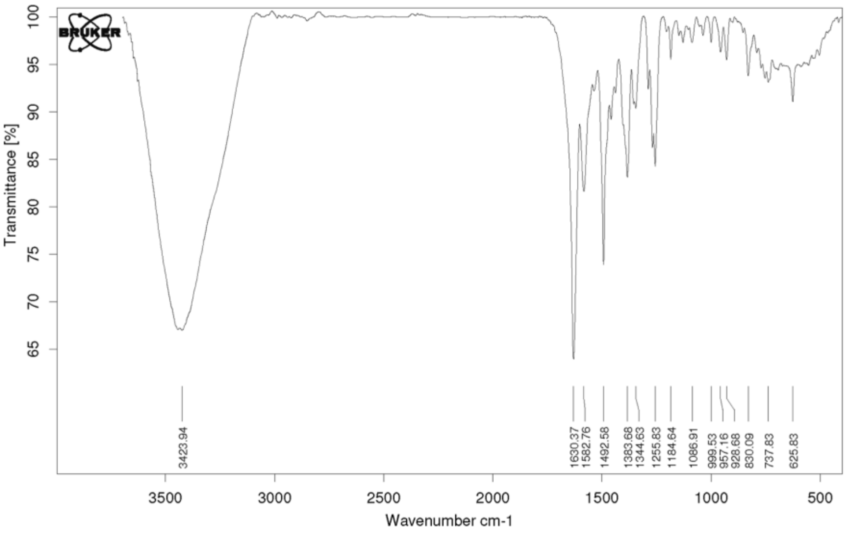

Figure S25 FTIR spectrum of [Ch][Nor] in KBr matrix.

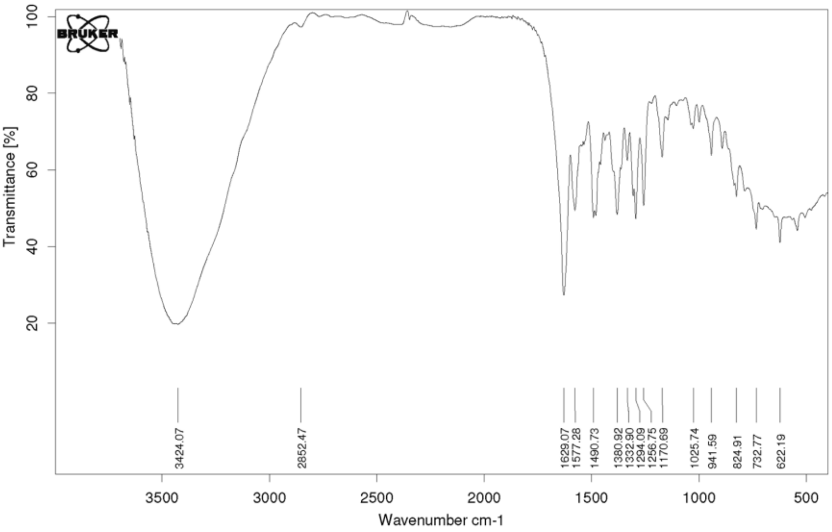

Figure S26 FTIR spectrum of [EMIM][Cip] in KBr matrix.

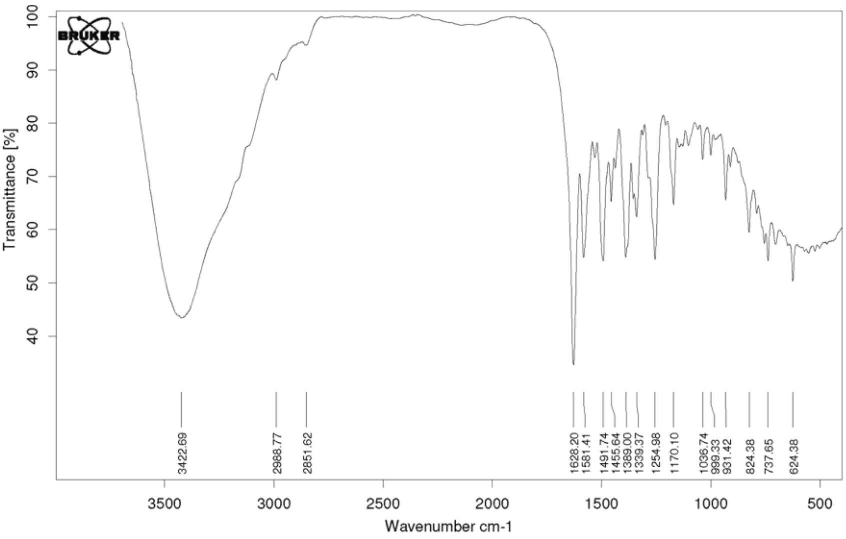

Figure S27 FTIR spectrum of [EMIM][Nor] in KBr matrix.

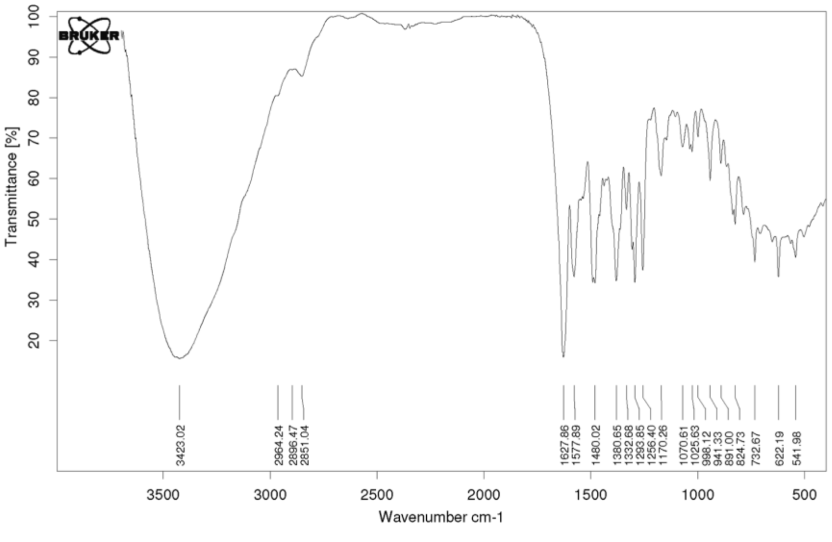

Figure S28 FTIR spectrum of [C_2OHMIM][Cip] in KBr matrix.

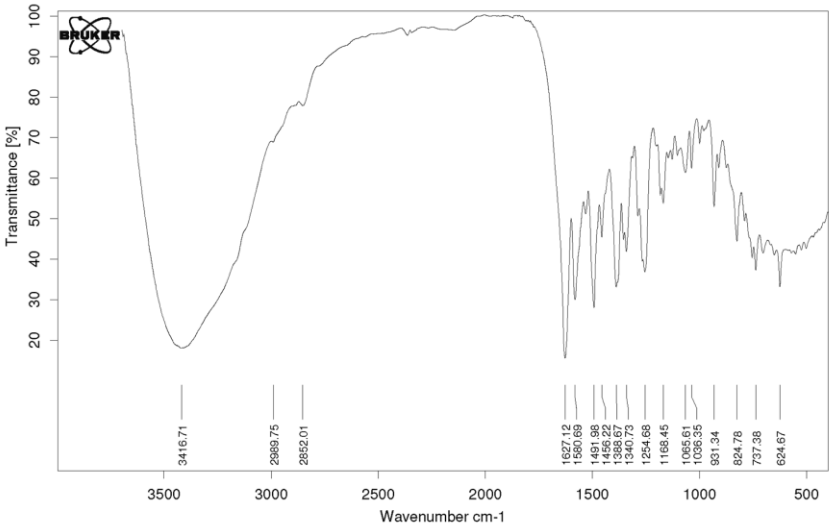

Figure S29 FTIR spectrum of [C2OHMIM][Nor] in KBr matrix.

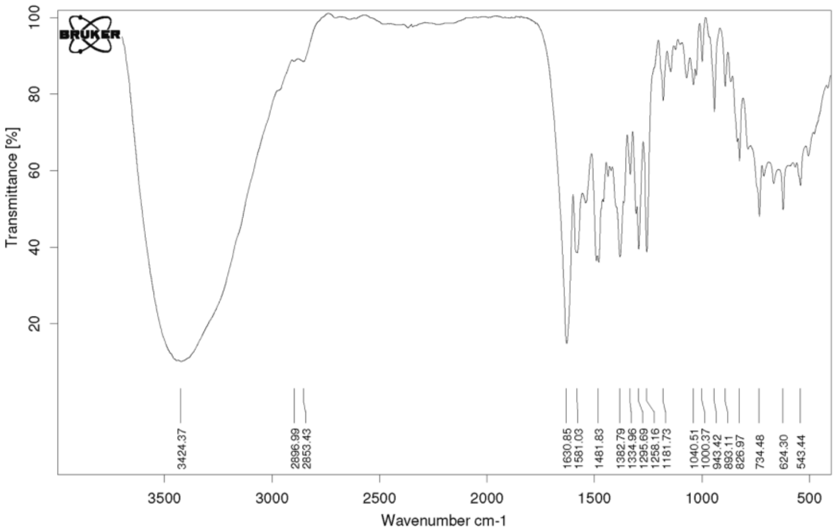

Figure S30 FTIR spectrum of [C2OHDMIM][Cip] in KBr matrix.

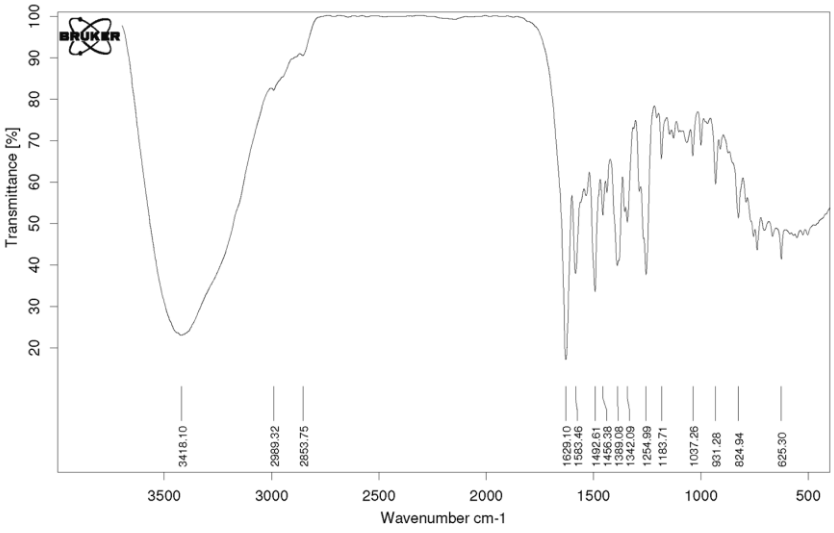

Figure S31 FTIR spectrum of [C2OHDMIM][Nor] in KBr matrix.

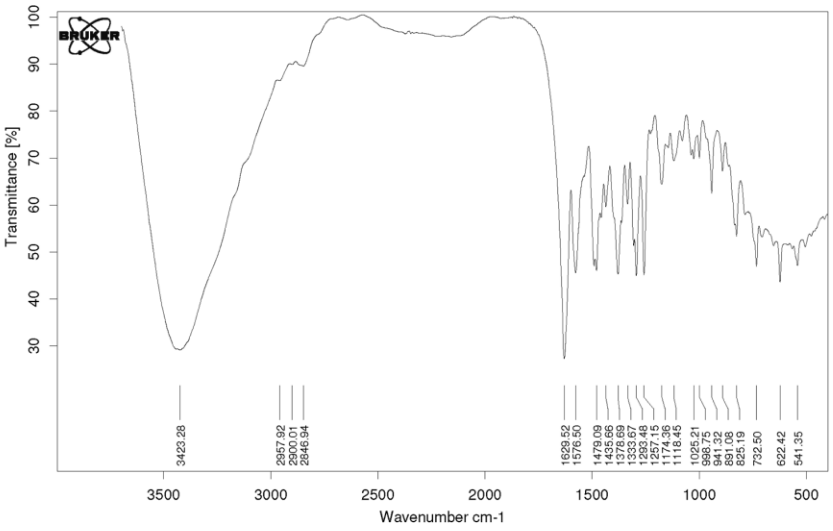

Figure S32 FTIR spectrum of [C_3OMIM][Cip] in KBr matrix.

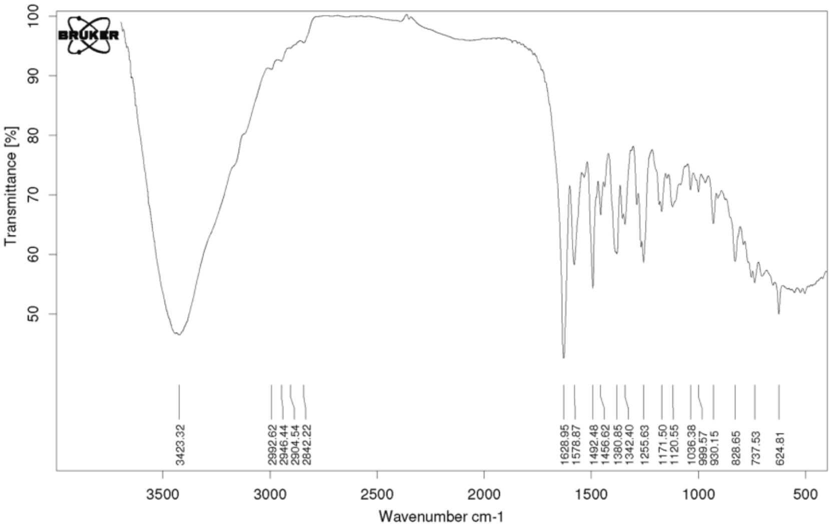

Figure S33 FTIR spectrum of [C_3OMIM][Nor] in KBr matrix.

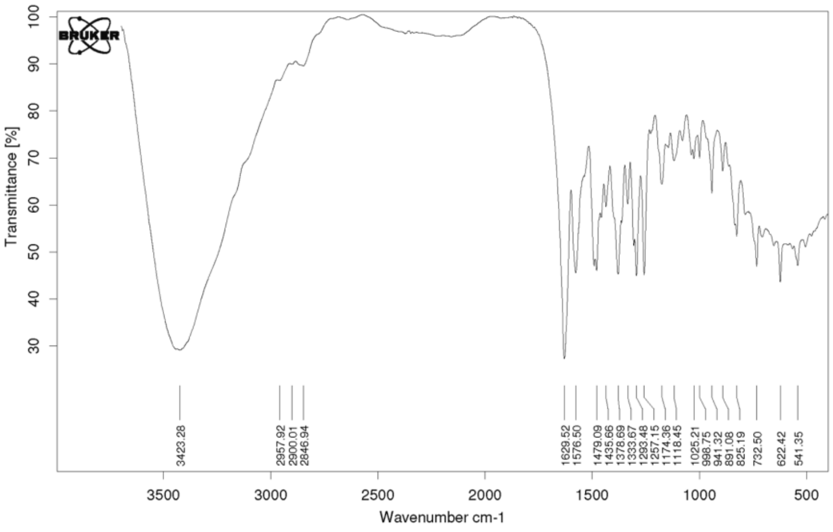

Figure S34 FTIR spectrum of [C_{16}Py][Cip] in KBr matrix.

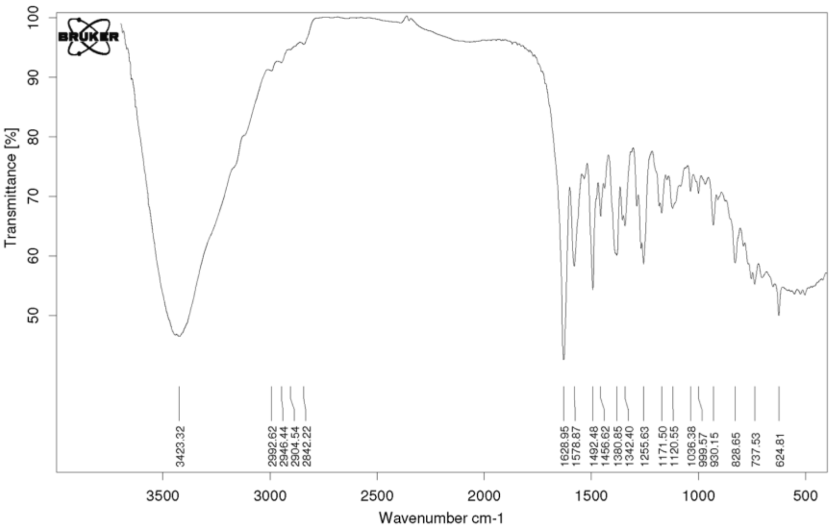

Figure S35 FTIR spectrum of [C16Py][Nor] in KBr matrix.

DSC thermograms

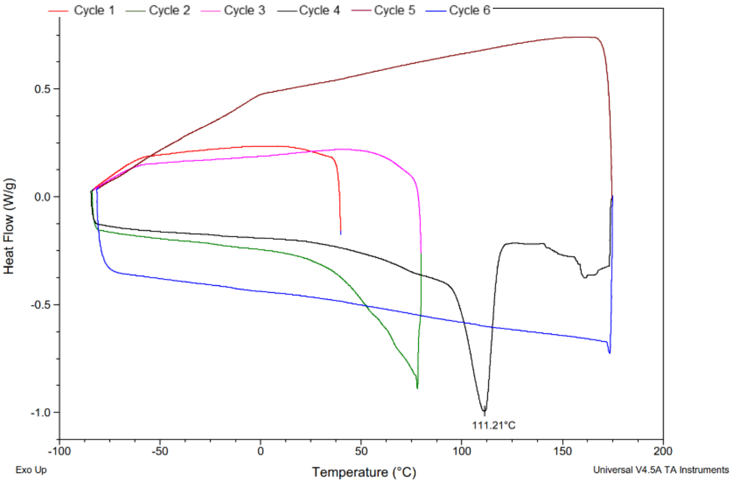

Figure S36 DSC thermogram of [Ch][Cip].

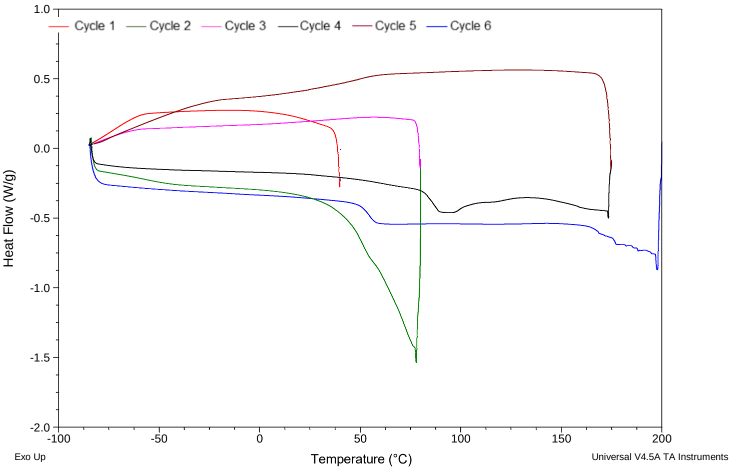

Figure S37 DSC thermogram of [Ch][Nor].

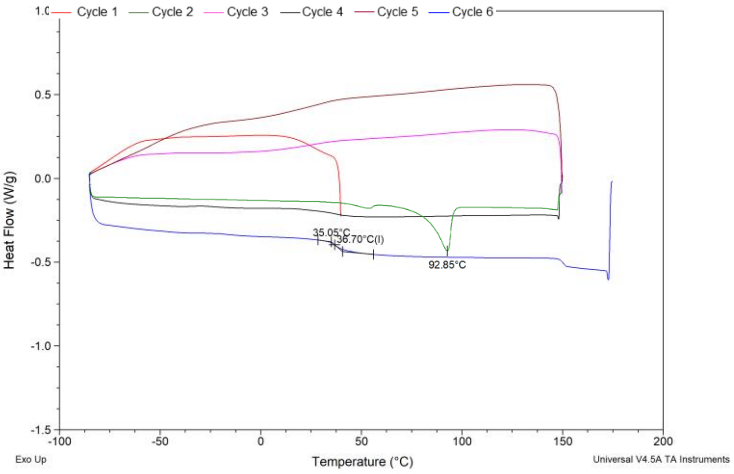

Figure S38 DSC thermogram of [EMIM][Cip].

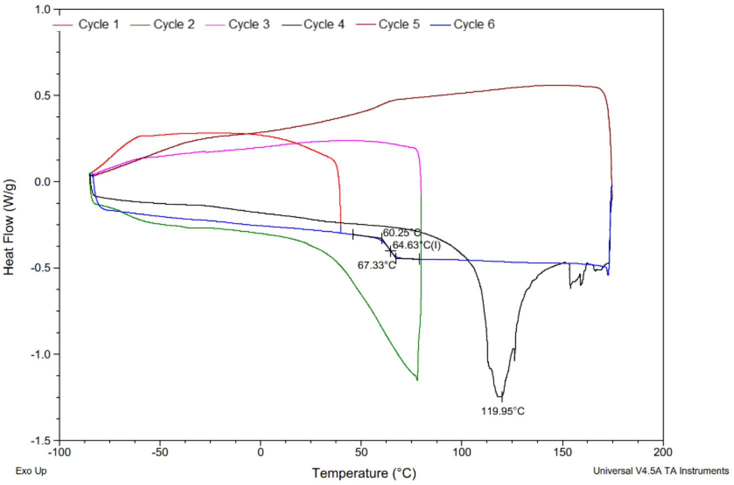

Figure S39 DSC thermogram of [EMIM][Nor].

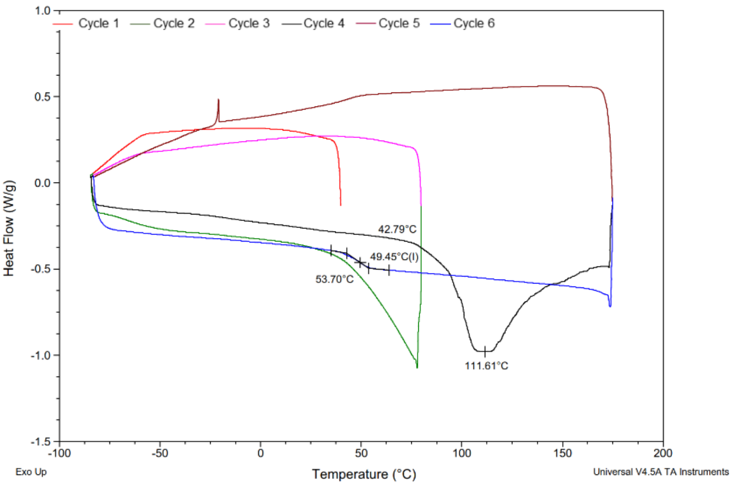

Figure S40 DSC thermogram of [C_2OHMIM][Cip].

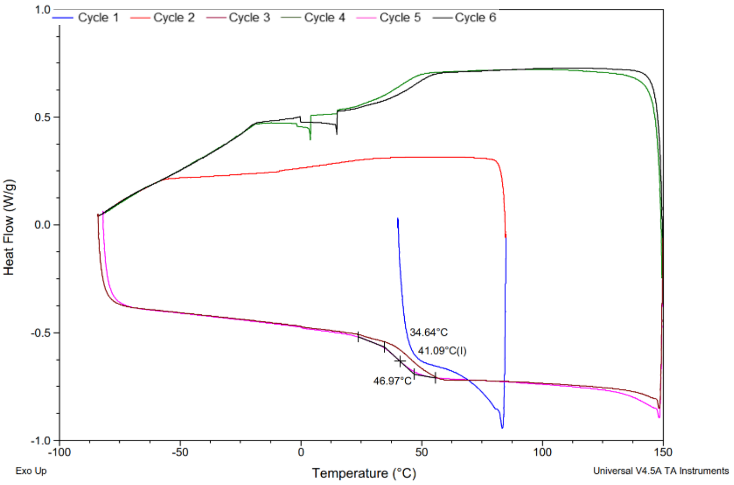

Figure S41 DSC thermogram of [C2OHMIM][Nor].

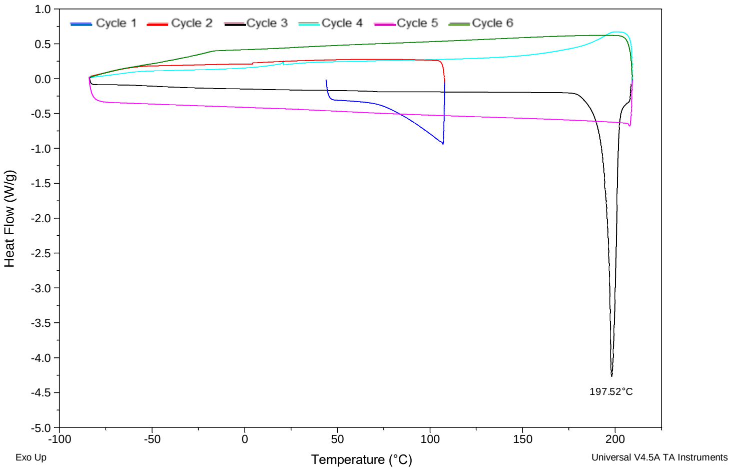

Figure S42 DSC thermogram of [C2OHDMIM][Cip].

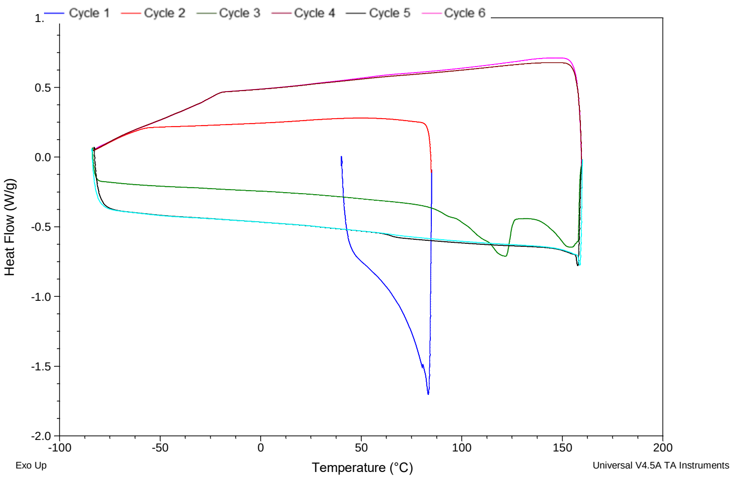

Figure S43 DSC thermogram of [C2OHDMIM][Nor].

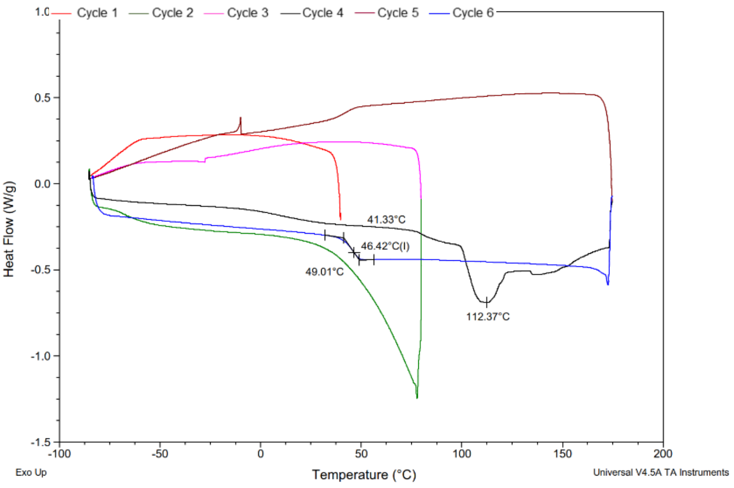

Figure S44 DSC thermogram of [C3OMIM][Cip].

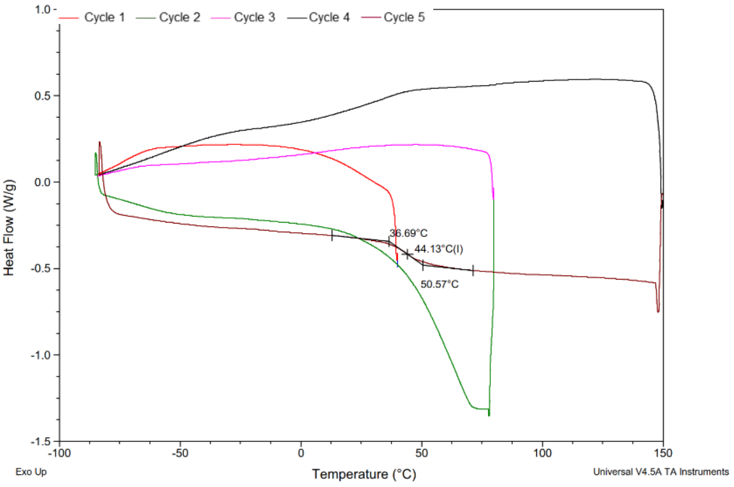

Figure S45 DSC thermogram of [C3OMIM][Nor].

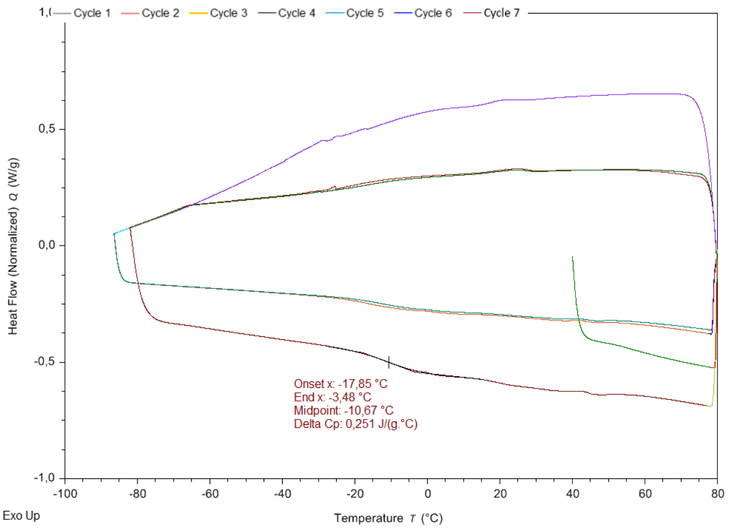

Figure S46 DSC thermogram of [C16Py][Cip].

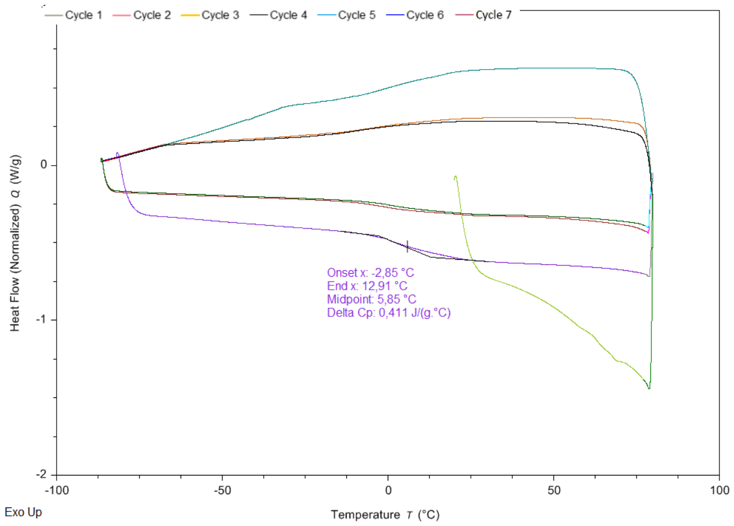

Figure S47 DSC thermogram of [C_{16}Py][Nor].

Determination of the solubility in water

In order to determine the solubility of the prepared organic salts based on ciprofloxacin and norfloxacin in water, samples were prepared in vials of 1.5 mL, by adding an excess amount of compound to the corresponding solvent. Vials were kept under vigorous stirring at 1400 rpm and controlled temperature (25 °C or 37 °C ± 0.1 °C), using of a *Thermomixer Comfort* (eppendorf 1.5 mL) and collected at different time periods. The samples were centrifuged in a *Biofuge 28 RS, Heraeus Sepatech*, for 10 minutes at 14000 rpm, in order to enhance physical separation of different phases. The quantification of the liquid phase was accomplished by UV-vis spectroscopy (model UV-1800 – Pharma-Spec spectrophotometer from Shimadzu), with quartz cells at a wavelength of 273 nm and 275 nm, which is the wavelength of the maximum absorbance of ciprofloxacin and norfloxacin in water, respectively. Triplicates were measured so that standard deviation could be calculated. A calibration curve was previously established for each compound in all of solvents used (for all curves $R^2 > 0.998$).

Table S1. Solubility in water of ciprofloxacin, ciprofloxacin hydrochloride, norfloxacin and organic salts based in fluoroquinolones (mean and standard deviation) at 25 °C and 37 °C.

**Compounds**	**Solubility (mg/mL)**	
	**25 °C**	**37 °C**
Ciprofloxacin	0.14 ± 0.01 [**Error! Bookmark not defined.**]<br>(0.09 ± 0.01) [**Error! Bookmark not defined.**]	0.18 ± 0.003 [**Error! Bookmark not defined.**]<br>(0.17 ± 0.01) [**Error! Bookmark not defined.**]
Ciprofloxacin.HCl	38.9 ± 0.8 [**Error! Bookmark not defined.**]<br>(38.4 ± 0.8 at 30 °C) [**Error! Bookmark not defined.**]	60.7 ± 1.2 [**Error! Bookmark not defined.**]<br>(58.4 ± 2.5 at 40 °C) [**Error! Bookmark not defined.**]
[Ch][Cip]	70.2 ± 3.6	101.0 ± 7.2
[EMIM][Cip]	34.5 ± 1.2	61.7 ± 3.9
[C_2OHMIM][Cip]	125.6 ± 7.8	259.2 ± 5.2
[C_2OHDMIM][Cip]	115.5 ± 8.4	147.4 ± 6.9
[C_3OMIM][Cip]	90.7 ± 10.0	169.7 ± 6.9

Norfloxacin	0.54 ± 0.02 [**Error! Bookmark not defined.**] (0.37 ± 0.02) [**Error! Bookmark not defined.**]	0.84 ± 0.02 [**Error! Bookmark not defined.**] (0.85 ± 0.04) [**Error! Bookmark not defined.**]
[Ch][Nor]	42.6 ± 6.8	76.7 ± 1.3
[EMIM][Nor]	25.3 ± 3.0	60.5 ± 3.7
[C_2OHMIM][Nor]	118.4 ± 3.2	223.8 ± 6.6
[C_2OHDMIM][Nor]	78.2 ± 7.3	89.1 ± 5.4
[C_3OMIM][Nor]	77.6 ± 6.4	138.3 ± 9.3

Critical micelle concentration

The critical micelle concentrations of the organic salts based on fluoroquinolones with cations bearing surfactant properties were calculated using ionic conductivity measurements. A CDM210 Radiometer Analytical conductimeter was used to measure the ionic conductivities (μS/cm) of surfactant solutions, in a glass cell containing a magnetic stirrer. In order to ensure the temperature control, a jacketed cell was used with a water bath controlled to ±0.01C. The cell temperature was measured by means of a PT100 platinum resistance sensor coupled to a Keithly 199 System DMM/Scanner.

The probe was previously calibrated against high accuracy mercury thermometers at different temperatures. In this method, a known amount of Milli-Q water was placed in a thermostated glass cell, at 25 °C, and the surfactant solution of known concentration was progressively added using a syringe. After each addition, the mixture was stirred to obtain a homogeneous solution, and the ionic conductivity was measured.

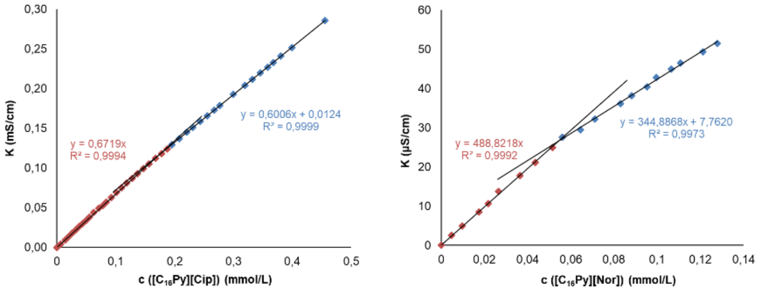

Figure S48. Plot of ionic conductivity in function of the concentration of [C_{16}Py][Cip] (left) and [C_{16}Py][Nor] (right).

Cytotoxicity of compounds

Cytotoxic activities of FQ-OSILs were studied on 3T3 cells previously acquired from DSMZ biobank. Cells were cultured in Dulbecco´s Modified Eagle´s medium: Nutrient Mix F-12 (DMEM/F-12) (Merck, Germany) supplemented with 10% FBS (Gibco, Grand Island, NY, USA), GlutaMAX™ (Gibco, Grand Island, NY, USA), 100 IU/mL penicillin, and 100 μg/mL streptomycin (Sigma, USA). For subculture, 3T3 cells were dissociated using trypsin-EDTA (Sigma, USA), splitted 1:5 ratio, and seeded into Petri dishes with 25 cm^2 of growth area. Culture medium was replaced every 2 days until cells reached the total confluence after 3–5 days of initial seeding. Cells were maintained in humidified atmosphere (95%), 5% CO_2 and 37 °C. Cytotoxicity was evaluated on 3T3 cells' viability after cells reached total confluence. Cells were treated with samples at 10 μM for 24 h. Samples effects were assessed by using MTT (Sigma, Germany), a colorimetric assay based on the conversion of tetrazolium salts to blue formazan products by active mitochondria. Results were expressed in percentage of control (%).

Antimicrobial activities

Antimicrobial activity of FQ-OSILs was evaluated against *Bacillus subtilis* (ATCC 6633) and *Staphylococcus aureus* (ATCC 25923) grown in Lysogeny broth (LB) and *Klebsiella pneumoniae* (ATCC 11296) grown in Tryptic soy broth. All mediums were obtained from Merck (Darmstadt, Germany). The antimicrobial activity was accompanied by optical density at 600 nm in order to verify the ability of compounds to inhibit the microorganisms growth (Synergy H1 Multi-Mode Microplate Reader, BioTek® Instruments, Vermont, USA). The IC_{50} was determined (0.001–10 nM) for the most potent samples (microorganism growth inhibition > 50% at 10 μM). Results were expressed in percentage of growth inhibition relative to the control (growth medium with microorganism).

Statistical analysis

Statistical analysis was performed using one-way analysis of variance (ANOVA) with Dunnett's multiple comparison of group means to determine significant differences relatively to control treatment. Results are presented as mean ± standard error of the mean (SEM). Differences were considered statistically significant at a level of 0.05 (p-value < 0.05). The determination of IC_{50} was performed by the analysis of non-linear regression by means of the equation:

$$y = \frac{100}{\left[1 + 10\left(X - log\left(IC_{50}\right)\right)\right]}$$

Calculations were performed using GraphPad v5.1 (GraphPad Software, La Jolla, CA, USA) software.

Table S2. IC_{50} values (nM) for the starting halide cations (0.001–10 μM) against the tested microorganisms. The values in parentheses represent the confidence intervals for 95%.

**Salt**	**IC_{50} (nM)**		
	S. aureus	***K. pneumoniae***	***B. subtilis***
[Ch]Cl	>10000	>10000	>10000
[EMIM]Br	>10000	>10000	>10000
[C_2OHmim]Cl	>10000	>10000	>10000
[C_2OHDMIM]Cl	>10000	>10000	>10000
[C_3Omim]Cl	>10000	>10000	>10000
[C_{16}Py]Cl	3249 (2922.0–3612.0)	2335 (2201.0–2477.0)	2548 (2390.0–2717.0)

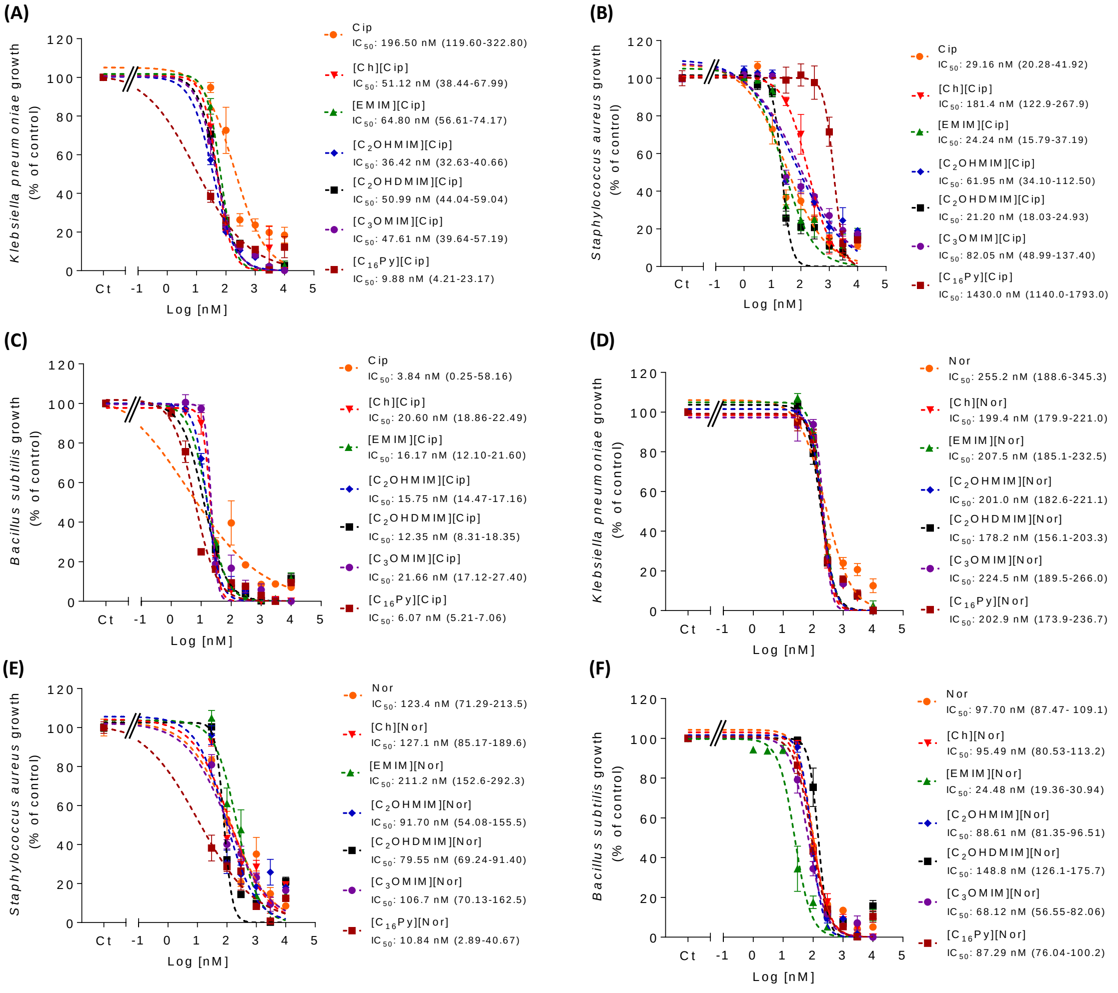

Figure S49. Dose-response curves of ciprofloxacin, norfloxacin and corresponding FQ-OSILs (0.001–10 nM) on *K. pneumoniae* (**A** and **D**), *S. aureus* (**B** and **E**) and *B. subtilis* (**C** and **F**) for IC_{50} determination. Values represent mean ± standard error of the mean (SEM) of at least three independent experiments carried out in triplicate.
